# Autophagy alleviates amiodarone-induced hepatotoxicity

**DOI:** 10.1007/s00204-020-02837-9

**Published:** 2020-07-10

**Authors:** Franziska Wandrer, Živa Frangež, Stephanie Liebig, Katharina John, Florian Vondran, Heiner Wedemeyer, Christian Veltmann, Tobias J. Pfeffer, Oren Shibolet, Klaus Schulze-Osthoff, Hans-Uwe Simon, Heike Bantel

**Affiliations:** 1 Department of Gastroenterology, Hepatology and Endocrinology, Hannover Medical School, Carl-Neuberg-Strasse 1, 30625 Hannover, Germany; 2 Institute of Pharmacology, University of Bern, Bern, Switzerland; 3 Department of Visceral and Transplantation Surgery, Hannover Medical School, Hannover, Germany; 4 Department of Cardiology and Angiology, Hannover Medical School, Hannover, Germany; 5 Department of Gastroenterology and Hepatology, Tel-Aviv Sourasky Medical Center and Sackler Faculty of Medicine, Tel-Aviv University, Tel-Aviv, Israel; 6 Interfaculty Institute of Biochemistry, University of Tübingen, Tübingen, Germany; 7 Department of Clinical Immunology and Allergology, Sechenov University, Moscow, Russia

**Keywords:** Amiodarone, Apoptosis, Autophagy, ER stress, Drug-induced liver injury, Keratin-18

## Abstract

Amiodarone is a widely used antiarrhythmic drug that can cause the development of steatohepatitis as well as liver fibrosis and cirrhosis. The molecular mechanisms of amiodarone-mediated liver injury remain largely unknown. We therefore analyzed amiodarone-mediated hepatocellular injury in patients with chronic heart failure, in primary hepatocytes and HepG2 cells. We found that amiodarone-treated patients with chronic heart failure revealed significantly higher serum levels of caspase-cleaved keratin-18, an apoptosis biomarker, compared to healthy individuals or patients not receiving amiodarone. Furthermore, amiodarone treatment of hepatocytes resulted in apoptosis associated with lipid accumulation and ER-stress induction. Liver cell steatosis was accompanied by enhanced de novo lipogenesis which, after reaching peak levels, declined together with decreased activation of ER stress. The decline of amiodarone-mediated lipotoxicity was associated with protective autophagy induction. In contrast, in hepatocytes treated with the autophagy inhibitor chloroquine as well as in autophagy gene (ATG5 or ATG7)-deficient hepatocytes, amiodarone-triggered toxicity was increased. In conclusion, we demonstrate that amiodarone induces lipid accumulation associated with ER stress and apoptosis in hepatocytes, which is mirrored by increased keratin-18 fragment serum levels in amiodarone-treated patients. Autophagy reduces amiodarone-mediated lipotoxicity and could provide a therapeutic strategy for protection from drug-induced liver injury.

## Introduction

Drug-induced liver injury (DILI) represents a major cause of liver failure and liver transplantation in western countries (Chalasani et al. 2015). DILI can occur as acute or chronic hepatitis, cholestasis or mixed cholestatic hepatitis (Chalasani et al. 2015). Twenty-six percent of DILI patients reveal liver steatosis, with macrovesicular steatosis as the dominant feature in 70% of cases (Kleiner et al. 2014; Rabinowich and Shibolet 2015). Drug-induced lipid accumulation in the liver is caused by increased de novo lipogenesis, reduced very low density lipoprotein secretion and/or drug-mediated mitochondrial dysfunction leading to impaired β-oxidation of fatty acids. Mitochondrial dysfunction further leads to increased production of reactive oxygen species (ROS) which elicits the peroxidation of fatty acids and thus the generation of pro-inflammatory cytokines. These events finally trigger the induction of steatohepatitis with the risk of developing liver fibrosis and cirrhosis (Begriche et al. 2011).

Amiodarone, an iodinated benzofuran derivative used for preventing and treating of atrial and ventricular arrhythmias, not only induces liver steatosis, but can also trigger the development of steatohepatitis and fibrosis (Lewis et al. 1989; Vassallo and Trohman 2007). Unlike most other cases of drug-induced liver injury, amiodarone-mediated liver injury can progress despite discontinuation of the drug, which might be explained by the long half-life and high accumulation of amiodarone in the liver (Lewis et al. 1989; Vassallo and Trohman 2007; Chang et al. 1999; Brien et al. 1987). Steatohepatitis induced by amiodarone was found to result in liver cirrhosis in up to 50% of cases (Rigas 1989; Farrell 2002; Raja et al. 2009). The underlying mechanisms of amiodarone-mediated liver damage, however, remain largely unclear.

It could be demonstrated that mice treated with amiodarone developed liver steatosis associated with increased mitochondrial ROS production and lipid peroxidation (Farrell 2002; Berson et al. 1998). Furthermore, endoplasmic reticulum (ER) stress was found to play an important role in amiodarone-mediated lipotoxicity (Erez et al. 2017; Lombardi et al. 2015). ER stress occurs when protein folding demand exceeds folding capacity, a situation which can be observed with increased accumulation of fatty acids in the liver. ER stress induction is followed by homeostatic signaling reactions, called unfolded protein response (UPR) (Ron and Walter 2007). UPR is transduced by different regulators, such as inositol-requiring enzyme-1 (IRE1)α and PKR-like ER kinase (PERK), both of which are activated by phosphorylation as well as by activating transcription factor-6 (ATF6) which is activated by intramembrane cleavage (Ron and Walter 2007). Activated IRE1α splices the mRNA of X-box binding protein-1 (XBP1) yielding the transcription factor sXBP1. Activated PERK phosphorylates eIF2α resulting in the preferential translation of ATF4 and activation of the transcription factor CHOP, which can also be activated by ATF6 (Ron and Walter 2007). CHOP up-regulates the expression of pro-apoptotic molecules of the death-receptor and mitochondrial pathway of apoptosis such as TRAIL-receptor(R)-2, PUMA and BIM and thereby contributes to ER-stress-mediated apoptosis (Cazanave et al. 2011; Puthalakath et al. 2007; Jung et al. 2015).

During apoptosis, caspases are activated and cleave various substrates including keratin-18 (K18), a major intermediate filament protein of hepatocytes. Caspase-cleaved K18 fragments are released from apoptotic hepatocytes and can be detected in sera from patients with liver diseases by an enzyme-linked immunosorbent assay (ELISA) detecting a caspase-generated neo-epitope of K18 (Bantel et al. 2004). In patients with non-alcoholic fatty liver disease (NAFLD) serum levels of caspase-cleaved K18 correlated with the extent of liver steatosis and liver injury (Joka et al. 2012; Tamimi et al. 2011). Moreover, detection of caspase-cleaved K18 could predict the requirement of liver transplantation or death in patients with DILI (Church et al. 2019). Thus, apoptosis plays an important role in drug-induced liver injury and outcome.

The reason why some patients with amiodarone treatment develop DILI and others not remains completely unclear. There is increasing evidence suggesting that autophagy protects from ER-stress induced cell death (Ogata et al. 2006). Autophagy regulates metabolic adaptation and maintains cellular homeostasis by removing aggregated and misfolded proteins as well as damaged organelles via cytosolic sequestration and subsequent lysosomal degradation. Autophagy gene (ATG)-related proteins thereby coordinate specific steps in autophagy induction. As an initial step, a double-membrane structure, called autophagosome, is formed. Its vacuole membrane then fuses with lysosomal membrane to deliver the cytoplasmic contents into autolysosomes, where they are degraded. During this process, microtubule-associated protein light chain 3 II (LC3-II), which is formed by phosphatidylethanolamine conjugation of LC3-I, translocates to the autophagosome membrane, thereby contributing to autophagosome formation. Autophagy can be detected by LC3-I/-II conversion as well as by decline of p62/sequestosome-1 (SQSTM1), a protein that is degraded by autophagy (Czaja 2016). It could be demonstrated that autophagy counter-regulates triglyceride accumulation in the liver (Singh et al. 2009). Vice versa, decreased ATG5 expression was associated with hepatocellular triglyceride accumulation in mice (He et al. 2013).

In the current study we analyzed the mechanisms of amiodarone-mediated liver injury. We found that amiodarone-treated patients with chronic heart failure revealed significantly higher serum levels of caspase-cleaved keratin-18 compared to healthy individuals or patients not receiving amiodarone. Furthermore, amiodarone was found to induce lipid accumulation in hepatocytes associated with increased ER-stress and apoptosis. Apoptosis of amiodarone-treated hepatocytes time-dependently declined which was paralleled by an increase of autophagy. Importantly, amiodarone-mediated cell death was significantly increased in hepatocytes deficient for the autophagy regulators ATG5 or ATG7. Our results, therefore, suggest that amiodarone-treated patients are at risk for ER-stress-mediated apoptotic liver injury and might benefit from autophagy-inducing therapeutic strategies.

## Materials and methods

### Cell culture of HepG2 and primary human hepatocytes

HepG2 cells were cultured in Dulbecco’s modified Eagle’s medium (Thermo Fisher Scientific, Waltham, MA, US), supplemented with 1 g/L of glucose, 10% fetal calf serum (Biochrom AG, Berlin, Germany), and 1% penicillin/streptomycin (Merck Millipore, Burlington, MA, US). Primary human hepatocytes (PHHs) were isolated as described (Kleine et al. 2014). Liver tissue was processed from different donors (*n* = 8) undergoing partial hepatectomy upon obtained written informed consent (approved by the Ethics Committee of Hannover Medical School, #252–2008). PHHs were cultured for 36 h in William’s medium E GlutaMAX (Thermo Fisher Scientific), supplemented with 1% penicillin/streptomycin, 10% fetal calf serum and 100 nM of dexamethasone for the first 12 h. HepG2 cells and PHHs were treated with 200 µM amiodarone (Sigma-Aldrich, St. Louis, MO, US) or the DMSO vehicle control.

### RNA preparation and quantitative real-time PCR

RNA was prepared using the RNeasy kit (Qiagen, Hilden, Germany) and cDNA was produced using the QuantiTect Reverse Transcription Kit (Qiagen). For expression analysis following primers from Sigma-Aldrich were used: *SREBP1c* (forward: 5′-GCGGAGCCATGGATTGCAC-3′; reverse: 5′-CTCTTCCTTGATACCAGGCCC-3′), *DGAT1* (forward: 5′-CTCAGATCCCACTGGCCTGG-3′; reverse: 5′-GTGGACGTACATGAGGACGGC-3′), *CHOP* (forward: 5′-CAAGAGGTCCTGTCTTCAGATGA-3′; reverse: 5′-TCT GTTTCCGTTTCCTGGTTC-3′). Expression of glyceraldehyde-3-phosphate dehydrogenase (*GAPDH*) mRNA was used as internal reference (QuantiTect Primer Assay, Qiagen). Real-time RT-PCR was performed in triplicates employing a SYBR Green RT-PCR Master Mix (Qiagen) and a QuantStudio system (Thermo Fisher Scientific) under the following conditions: 40 cycles of 95 °C for 10 s, 5 s at 55 °C and 240 s at 72 °C. Data were analyzed using the comparative (ΔΔ*C*_*T*_) method with normalization to *GAPDH* expression.

### Detection of cell death, ER-stress and steatosis in hepatocytes

HepG2 cells were seeded on poly-L-lysine (Sigma-Aldrich) coated coverslips and incubated with amiodarone or vehicle control. The cells were fixed with 4% paraformaldehyde and detection of TUNEL reactivity was performed using the In-Situ Cell Death Detection Kit according to the manufacturer's instructions (Roche, Basel, Switzerland). After repeated washings, nonspecific binding was blocked with 5% goat serum in PBS for 10 min. Cells were then incubated with an anti-cleaved caspase-3 antibody (Cell Signaling, Danvers, MA, US) for 1 h. Following washing, cells were incubated with the Cy3-labeled secondary antibody (Jackson Laboratory, Bar Harbor, ME, US) for an additional hour. After final washing in PBS, cells were counterstained with DAPI (Thermo Fisher Scientific) and covered with fluorescence-mounting medium. Cells were microscopically imaged at 200× magnification (Olympus BX51 and Cell Software, Shinjuku, Japan).

For immunocytochemical detection of activated IRE1α, cells were incubated with a pIRE1α antibody (Abcam, Cambridge, UK) for 1 h after blocking with 5% goat serum. After washing, cells were incubated with a Cy3-labeled secondary antibody (Jackson Laboratory) for an additional hour. Following final washing, cells were counterstained with DAPI (Thermo Fisher Scientific) and covered with fluorescence-mounting medium. For triglyceride detection, Oil Red O staining of cells was performed according to the protocol of the manufacturer (Sigma-Aldrich). Triglyceride quantification was performed at 400x magnification using Image J software (Schindelin et al. 2012).

### Immunoblotting

Cell lysates were separated under reducing conditions on 12 or 15% SDS–polyacrylamide gels and electroblotted to a polyvinylidene difluoride membrane. After 1 h of blocking in 5% nonfat dry milk powder in TBST, membranes were incubated with primary antibodies over night at 4 °C. Polyclonal anti-actin and monoclonal anti-p62/SQSTM1 antibodies were from Santa Cruz (Santa Cruz Biotechnology, Dallas, TX, US), monoclonal anti-ATG5 (7C6) and monoclonal anti-LC3 antibodies from NanoTools (Teningen, Germany), monoclonal anti-ATG7 (D12B11), polyclonal anti-cleaved caspase-3, monoclonal anti-CHOP, polyclonal anti-p-eIF2α (Ser51), polyclonal anti-eIF2α, polyclonal anti-p62/SQSTM1 and monoclonal anti-GAPDH antibodies were from Cell Signaling. Polyclonal anti-pIRE1α (S724) antibody was used from Abcam (Cambridge, UK), polyclonal anti-caspase-3 was from R&D (Minneapolis, MN, US) and polyclonal anti-LC3B antibody from Novus Biologicals (Littleton, CO, US). Monoclonal anti-α-tubulin antibody was purchased from Sigma-Aldrich.

### CRISPR/Cas9-mediated downregulation of ATG5 and ATG7

Gene knockouts for human *ATG5* (Gene ID: 9474) or *ATG7* (Gene ID: 10533) in HepG2 cells were achieved using CRISPR/Cas9 technology. Specific guide RNAs (gRNA) were designed using publicly available CRISPOR software (http://crispor.tefor.net). The *ATG5* knockout was stably prepared using the LentiCRISPR v2 vector (gift from Feng Zhang, Addgene plasmid #52961) (Sanjana et al. 2014), while the *ATG7* knockout was conditionally prepared using the doxycycline-inducible FgH1tUTG and FUCas9Cherry plasmids (gift from Marco Herold, Addgene plasmid #70183 and #70182, respectively) (Aubrey et al. 2015). Sequences used for gRNA construction: *ATG5* Fw 5′-CACCGGTGCTTCGAGATGTGTGGTT-3′, Rev 5′-AAACAACCACACATCTCGAAGCACC-3′, *ATG7*: Fw 5′-TCCCGAAGCTGAACGAGTATCGGC-3′ and Rev 5′-AAACGCCGATACTCGTTCAGCTTC-3’.

Recombinant lentiviruses were produced after calcium phosphate transfection together with the envelope vector PMD2.G and the packaging vectors psPAX2 (provided by Dr. D. Trono, University of Geneva, Switzerland) in 293 T cells. Viruses were harvested 24 h after transfection, filtered through a 0.22 µm membrane (Merck Millipore), and stored at − 80 °C until use. HepG2 cells were transduced with the desired virus in the presence of 8 µg/mL polybrene. Cell clones with a constitutive *ATG5* knockout were selected in the presence of 2 µg/mL puromycin for 2 weeks. Cells with the inducible *ATG7* knockout were FACS-sorted according to their GFP (FgH1UTG) and mCherry (FUCas9Cherry) expression. Control HepG2 lines were prepared in parallel by infection with non-gRNA coding lentiviruses. Before the experiments, the inducible *ATG7* knockout and control cells were treated with 2.5 µg/mL doxycycline (Thermo Fisher Scientific) for 5 days.

### Detection of caspase activity

Caspase-3/-7 activity was measured in triplicate by a luminescent substrate assay (Caspase-Glo; Promega, Mannheim, Germany). Cell extracts were diluted in 50 mM Tris–HCl (pH 7.4), 10 mM KCl, and 5% glycerol to reach a final protein concentration of 0.1 mg/mL. Then, 10 µL of the extracts were incubated with 10 µL of the caspase substrate DEVD-luciferin and luciferase reagent for 2 h at room temperature. Luminescence of the samples was measured using a luminometer (LB 960, Berthold Technologies, Bad Wildbad, Germany), yielding relative light units (RLU).

### Detection of cell viability

Cell viability was measured using the RealTime-Glo™ MT Cell Viability Assay (Promega). HepG2 control, *ATG5* or *ATG7* knockout cells were plated in white 96-well assay plates (5 × 10^3^ cells/well). The MT Cell Viability Substrate and NanoLuc® Enzyme were added to the cultures together with 200 µM of amiodarone or with the drug vehicle control. The luminescence signal was measured at 6 h using the GloMax Explorer Multimode Microplate Reader (Promega). Results are presented as percentage (mean ± SEM) of amiodarone-treated cells normalized to DMSO-treated control cells from two separate experiments performed with 4–5 replicates.

### Serological detection of caspase-cleaved keratin-18

For quantitative measurement of the caspase-generated neo-epitope of K18, we used the M30-Apoptosense ELISA (Peviva, Bromma, Sweden) according to the manufacturer’s instructions as described (Bantel et al. 2004; Joka et al. 2012). Sera from patients with chronic heart failure (*n* = 15) either treated (*n* = 6) or non-treated (*n* = 9) with amiodarone (200 mg/day) as well as from healthy persons (*n* = 13) were analyzed in duplicate for caspase-mediated K18 cleavage fragments. At the time of blood analyses patients received amiodarone for at least 4 months. Patients with or without amiodarone treatment revealed no significant differences in age (75.3 ± 3.5 vs. 72.2 ± 3.2 years), sex (83.3% vs. 77.8% of male), type 2 diabetes (33.3% vs. 33.3%), body mass index (BMI; 27.4 ± 1.5 vs. 28.8 ± 1.0) as well as in the use of lipid lowering or anti-coagulative drugs. The study was performed according to the Ethics Committee of Hannover Medical School.

### Statistical analyses

Statistical analyses of the cell culture experiments were performed using paired *t*-test (equal distribution) or one-sample *t*-test (mRNA expression compared to untreated control). For statistical analysis of AST/ALT and caspase-cleaved K18 serum levels unpaired *t*-test (equal distribution) and of clinical characteristics Mann Whitney’s *U* test (non-equal distribution) were performed using GraphPad Prism 5.0 (GraphPad Software, La Jolla, CA, US). Data are presented as mean ± standard error of the mean (SEM). A *p*-value of less than 0.05 was considered significant.

## Results

### Amiodarone-treated patients with chronic heart failure reveal increased caspase activation, which is mirrored by amiodarone treatment of isolated hepatocytes

**Fig. 1:**
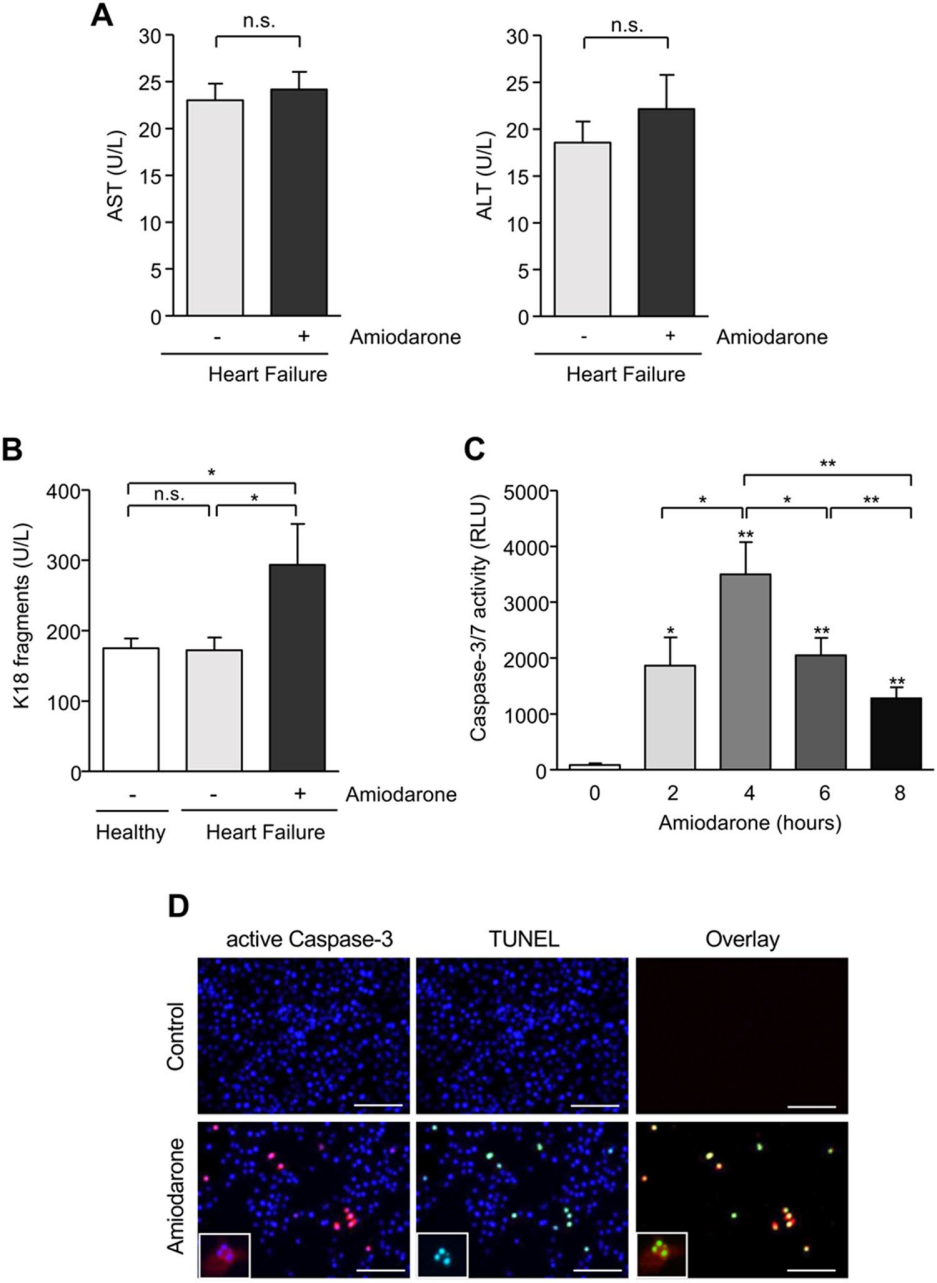
Amiodarone treatment results in liver cell apoptosis. **a** Detection of serum AST and ALT levels in chronic heart failure patients with or without amiodarone treatment. Patients with chronic heart failure treated with amiodarone revealed slightly elevated aminotransferase levels compared to patients without amiodarone treatment, which was, however, not significant. **b** Detection of caspase-cleaved keratin-18 (K18) in sera from chronic heart failure patients. Treatment of chronic heart failure patients with amiodarone resulted in significantly higher serum levels of caspase-cleaved K18 compared to patients not receiving amiodarone or healthy persons. **c** Amiodarone time-dependently induces caspase-3/-7 activity in HepG2 cells. Cells were treated for the indicated time with amiodarone (200 µM), before caspase activity was measured by a luminometric substrate assay. Data were obtained from 5 independent experiments. **d** Immunocytochemistry of caspase-3 activation and TUNEL staining after amiodarone treatment of HepG cells. Cells were either left untreated or incubated for 4 h with 200 µM amiodarone. Merged images were produced by overlaying caspase-3 (red), TUNEL (green) and nuclear DAPI (blue) staining. Bars = 100 µm. Significances indicated above the bars refer to control. *p < 0.05, **p < 0.01, n.s. = non-significant (colour figure online)

In initial experiments we asked whether amiodarone treatment of patients with chronic heart failure results in apoptotic liver injury. We first compared alanine aminotransferase (ALT) and aspartate aminotransferase (AST) levels in sera from patients with chronic heart failure either receiving (*n* = 6) or not receiving (*n* = 9) amiodarone treatment (Fig. 1a). We found only marginally and within the normal range elevated AST (24.2 ± 1.9 U/L) and ALT (22.2 ± 3.6 U/L) levels in amiodarone-treated patients compared to those not receiving amiodarone (23.0 ± 1.8 U/L and 18.6 ± 2.2 U/L) which was, however, not significant. Next, we analyzed the serum levels of caspase-cleaved K18. The intermediate filament protein K18 is expressed in hepatocytes and cleaved during apoptosis by caspases. A cleavage-generated K18 fragment is released in the blood stream and has previously been established as a reliable biomarker of apoptotic liver injury (Bantel et al. 2004; Joka et al. 2012; Tamimi et al. 2011). Using a specific ELISA (Fig. 1b), we interestingly found that heart failure patients receiving amiodarone revealed significantly higher serum levels of caspase-cleaved K18 (*n* = 6; 293.6 ± 58.4 U/L) compared to patients without amiodarone therapy (*n* = 9; 172.1 ± 17.9 U/L) or healthy control individuals (*n* = 13; 174.8 ± 14.0 U/L). Heart failure patients without amiodarone treatment showed similar levels of caspase-cleaved K18 as healthy controls, indicating that heart failure per se did not influence liver injury in those patients. Moreover, chronic heart failure has been previously associated with necrotic rather than apoptotic cell death (Bechmann et al. 2010). These data, therefore, indicate that amiodarone treatment of patients with chronic heart failure results in apoptotic liver injury, which can be detected with high sensitivity using caspase-cleaved K18.

To further investigate the role of apoptosis for amiodarone-mediated liver toxicity, we analyzed the activation of effector caspase-3/-7 in amiodarone-treated HepG2 cells using a luminometric substrate assay. Initial dose-finding experiments revealed effective caspase activation with 200 µM amiodarone (data not shown), a concentration which was found to cause hepatotoxicity in previous studies (Erez et al. 2017; Fromenty 1990b) and, therefore, used for further experiments. Compared to untreated control, treatment of HepG2 cells with amiodarone significantly increased caspase-3/-7 activation with a maximum at 4 h (3496.7 ± 576.8 RLU vs. 83.6 ± 31.4 RLU; *p* < 0.01), followed by a decline at 6 and 8 h of treatment (2042.8 ± 314.1 RLU and 1281.8 ± 196.6 RLU; Fig. 1c). Double staining for active caspase-3 and TUNEL reactivity revealed that caspase activation in HepG2 cells was indeed associated with apoptosis following 4 h of amiodarone treatment (Fig. 1d).

### Amiodarone induces lipid accumulation in hepatocytes

**Fig. 2:**
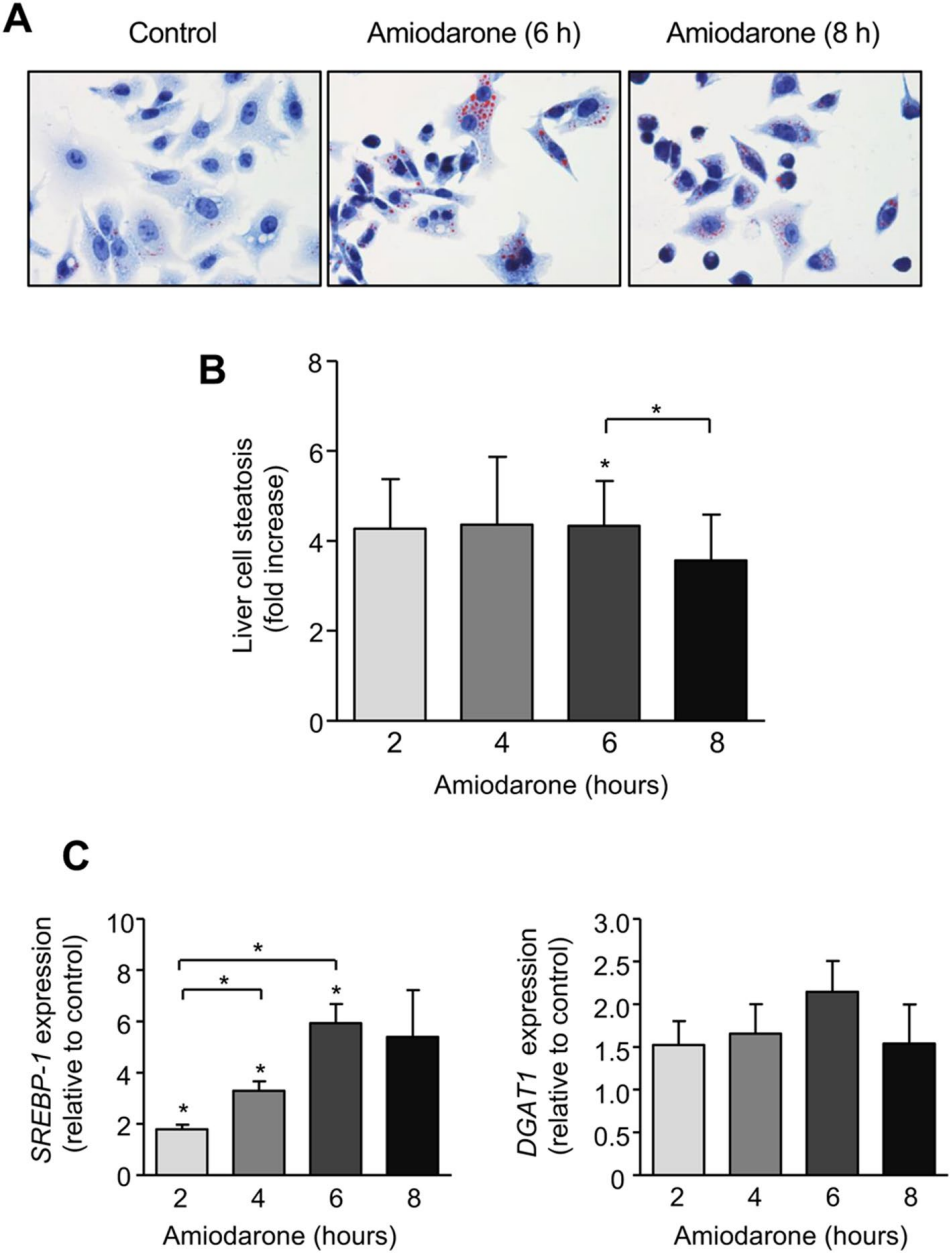
Amiodarone induces hepatocyte steatosis and increased expression of lipogenic regulators. **a**, **b** Triglyceride accumulation in HepG2 cells, as assessed by Oil Red O staining, was induced after 2–6 h of amiodarone treatment (200 µM) followed by a decline after 8 h. **c** Transcription of the lipogenic regulators *SREBP1c* and *DGAT1* was induced by amiodarone treatment of HepG2 cells with peak levels at 6 h and a decrease at 8 h. Data were obtained from 3 independent experiments. Significances indicated above the bars refer to control. *p < 0.05

Since lipid accumulation can trigger pathways of apoptotic cell death, we next analyzed amiodarone-mediated hepatocyte steatosis by Oil Red O staining of triglycerides. We found that amiodarone induced lipid accumulation in HepG2 cells, which was most strongly pronounced after 4 and 6 h of incubation (4.4 ± 1.5 and 4.3 ± 1.0 fold increase compared to untreated control; Fig. 2a, b), similar to our observation of maximal caspase activation (Fig. 1c). Hepatocyte steatosis significantly (*p* < 0.05) declined after 8 h of amiodarone treatment (3.6 ± 1.0 fold increase compared to untreated control) compared to 6 h of amiodarone incubation (Fig. 2a, b).

Based on these observations, we analyzed the potential effect of amiodarone on de novo lipogenesis in hepatocytes. We found that amiodarone induced mRNA expression of sterol-regulatory element-binding protein-1c (*SREBP1c*), a major transcription factor of lipogenesis, in a similar time frame as observed for amiodarone-induced lipid accumulation. *SREBP1c* expression significantly (*p* < 0.05) increased from 2 h to 4 and 6 h of amiodarone incubation (5.9 ± 0.7 fold increase compared to untreated control) followed by a decline at 8 h of treatment (Fig. 2c). Since diacylglycerol-acyltransferase-1 (DGAT1) is a critical regulator of triglyceride formation, we also assessed the transcription of this enzyme in amiodarone-treated vs. untreated HepG2 cells. Like with *SREBP1c*, we found an increase in *DGAT1* mRNA peaking at 6 h after amiodarone treatment (2.1 ± 0.4 fold increase compared to control) (Fig. 2c). Thus, amiodarone-induced lipid accumulation in hepatocytes is associated with enhanced mRNA expression of the lipogenic regulators *SREBP1c* and *DGAT1*.

### Amiodarone induces endoplasmic reticulum stress in hepatocytes

**Fig. 3:**
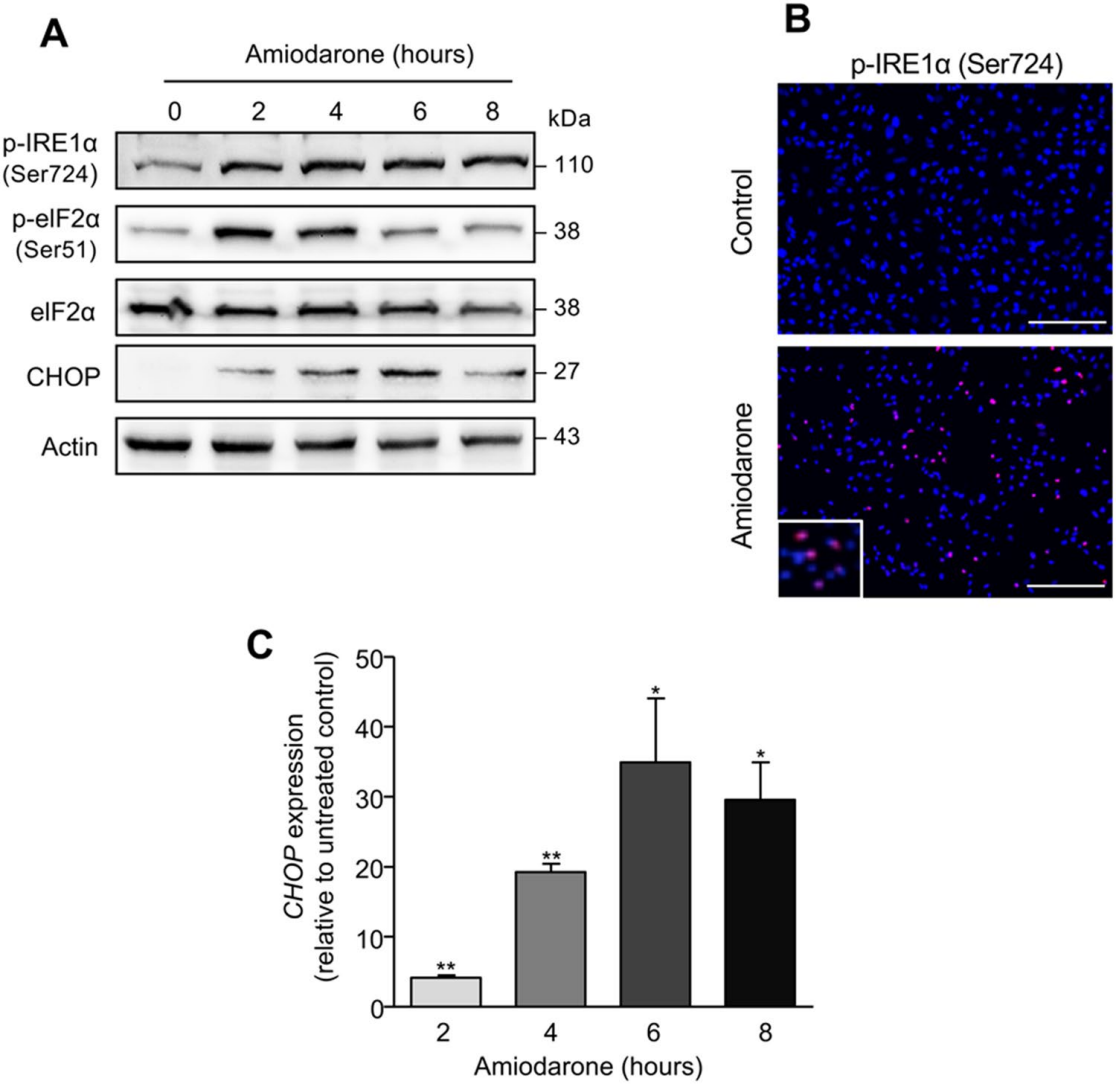
Amiodarone induces ER stress. **a** Western blot analysis demonstrated that amiodarone treatment (200 µM) of HepG2 cells results in the phosphorylation of IRE1α and eIF2α as well as in increased expression of the transcription factor CHOP. Phosphorylation of eIF2α and CHOP expression increases up to 4–6 h followed by a decline at 8 h of treatment. **b** Immunocytochemical detection of phosphorylated and hence activated IRE1α in HepG2 cells treated for 4 h with amiodarone further confirms ER stress induction. **c** Quantification of *CHOP* mRNA after amiodarone treatment of HepG2 cells compared to untreated control cells. Data were obtained from 4 independent experiments. Bars = 100 µm. *p < 0.05, **p < 0.01

To further explore the pathways of amiodarone-mediated lipotoxicity, we analyzed the effect of amiodarone on ER-stress induction. We found that amiodarone treatment of HepG2 cells led to a time-dependent phosphorylation and, hence, activation of the ER-stress regulator IRE1α which was most pronounced after 4 h, as demonstrated by Western blot analysis (Fig. 3a). Activation of IRE1α was confirmed by immunocytochemistry which showed an increased number of HepG2 cells positive for phosphorylated IRE1α staining after 4 h of amiodarone treatment compared to untreated control cells (Fig. 3b). Furthermore, enhanced IRE1α activation was associated with a decreased cell number (Fig. 3b).

In addition to IRE1α, we analyzed eIF2α, a subunit of the eukaryotic translation initiation factor 2, which is phosphorylated by PERK during the unfolded protein response resulting in a global downregulation of protein synthesis. Phosphorylation of eIF2α was visible after 2–4 h of amiodarone treatment and thereafter decreased (Fig. 3a)*.* In accordance, expression of the downstream transcription factor CHOP was strongly induced at the protein (Fig. 3a) and mRNA level (Fig. 3c) by amiodarone treatment. *CHOP* transcripts reached a peak expression at 6 h of amiodarone incubation (34.9 ± 9.1 fold increase relative to untreated control, *p* < 0.05) and thereafter declined (Fig. 3a, c).

### Amiodarone triggers protective autophagy in hepatocytes

**Fig. 4:**
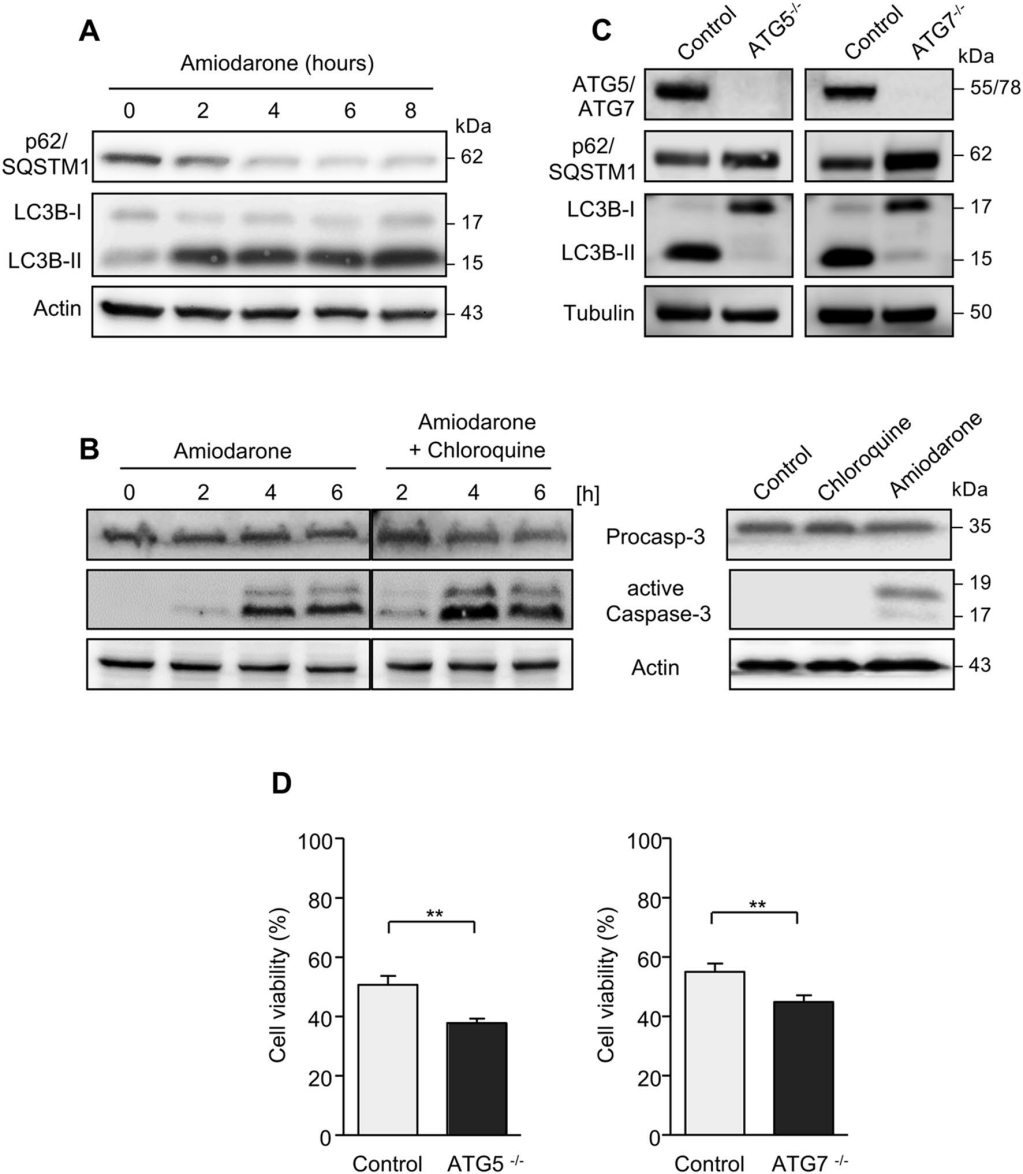
Amiodarone treatment triggers autophagy. **a** Western blot analysis revealed decreased p62/SQSTM1 expression and increased LC3B-I/II conversion in HepG2 cells treated with amiodarone for 2–8 h. **b** Compared to amiodarone treatment alone, pretreatment of HepG2 cells with the autophagy inhibitor chloroquine increased caspase-3 activation at 4 and 6 h of amiodarone treatment (left panel). The vertical lines indicate juxtaposition of non-adjacent lanes from the same blot. In contrast to amiodarone, chloroquine itself did not induce caspase-3 activation (right panel). **c** Compared to the corresponding control cells, *ATG5*- or *ATG7*-deficient HepG2 cells revealed decreased autophagy as shown by p62/SQSTM1 stabilization and lack of LC3B-I/-II conversion in Western blot analyses. **d** *ATG5*- or *ATG7*-deficiency resulted in enhanced cell death of HepG2 cells after 6 h of amiodarone treatment as compared to control cells. **p < 0.01

Since lipid accumulation in hepatocytes is associated with autophagy induction, we asked whether amiodarone treatment of HepG2 cells also results in enhanced autophagy. Amiodarone indeed induced autophagy, as indicated by a decrease of p62/SQSTM1 and increased LC3B-I/II conversion, which are two events required for autophagosome formation (Fig. 4a). Compared to amiodarone treatment alone, combined treatment of HepG2 cells with amiodarone and the autophagy inhibitor chloroquine for 4 or 6 h resulted in enhanced caspase-3 activation (Fig. 4b). In contrast to amiodarone, treatment with chloroquine alone did not induce caspase-3 activation (Fig. 4b).

To further analyze a potentially protective role of autophagy in amiodarone-mediated liver cell toxicity, we generated HepG2 cells deficient for autophagy-regulating genes, such as *ATG5* and *ATG7*, using CRISPR/Cas-9 technology. As demonstrated by Western blot analysis (Fig. 4c), HepG2 cells deficient for *ATG5* or *ATG7* revealed a lower level of autophagy, as indicated by a p62/SQSTM1 stabilization and reduced LC3B-I/II conversion compared to the respective control cells. Amiodarone treatment of *ATG5*-deficient HepG2 cells for 6 h resulted in significantly (*p* < 0.01) lower cell viability compared to control cells (Fig. 4d). Similarly, also *ATG7* deficiency resulted in enhanced cell death of HepG2 cells after 6 h of amiodarone treatment as compared to the respective control cells (Fig. 4d). Thus, amiodarone-mediated toxicity was attenuated by its autophagy-inducing property.

### Amiodarone-induced ER stress, apoptosis and autophagy in primary human hepatocytes

**Fig. 5:**
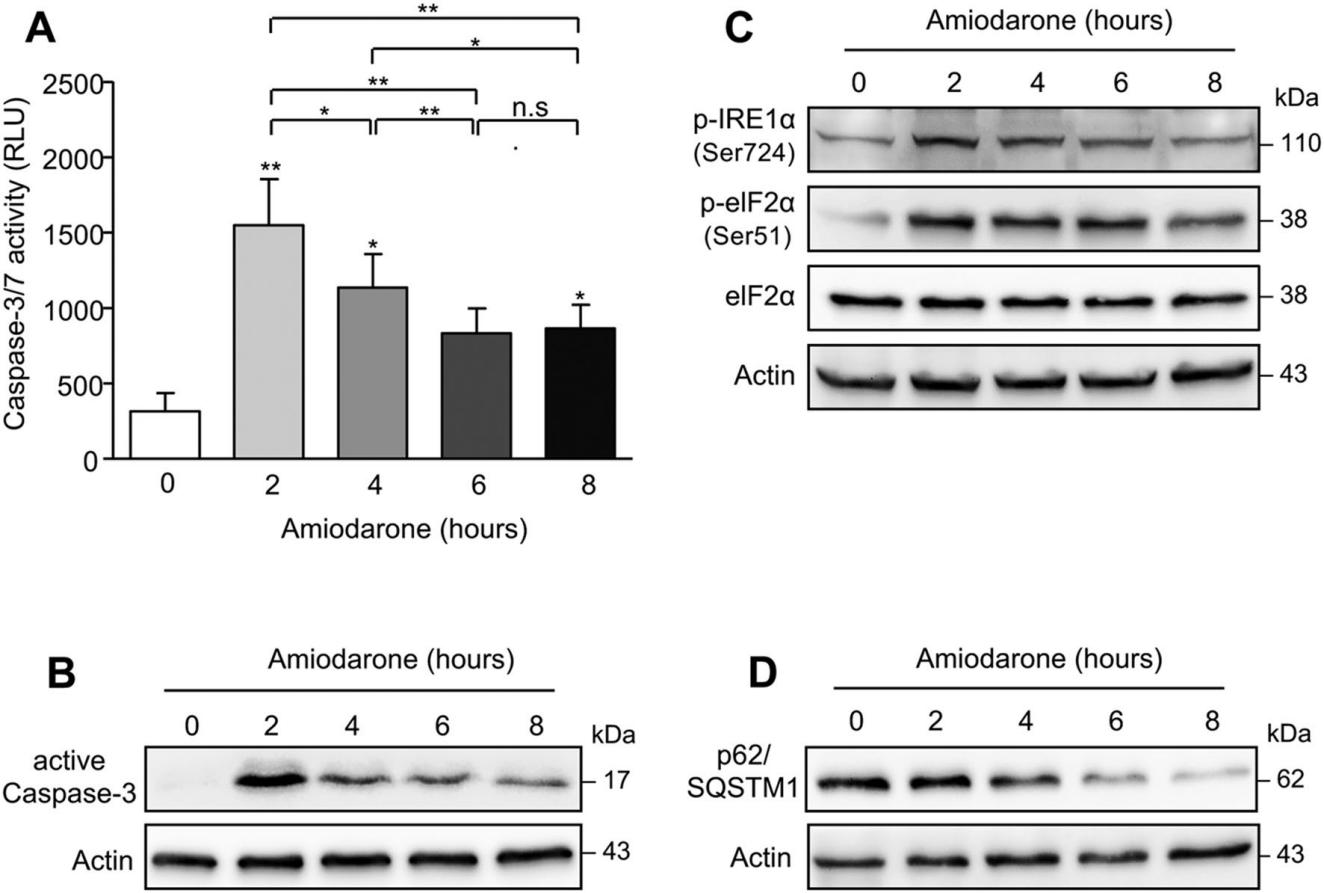
Amiodarone-induced apoptosis, ER-stress and autophagy in primary human hepatocytes. **a** Treatment of PHHs (*n* = 8 donors) with amiodarone (200 µM) for 2 h significantly increased caspase-3/-7 activity followed by a significant decline at 4–8 h of treatment. **b** Western blot analysis confirmed a significant increase of activated caspase-3 after 2 h of PHH treatment with amiodarone, which then decreased at 4–8 h of treatment. **c** ER stress is triggered in PHHs by amiodarone, indicated by phosphorylation of IRE1α and eIF2α, which peaked at 2 h of treatment and thereafter declined. **d** Autophagy was induced at 4–8 h of amiodarone treatment of PHHs as indicated by decreased p62/SQSTM1. Significances indicated above the bars refer to control. *p < 0.05, **p < 0.01

To further verify our results obtained in HepG2 cells, we analyzed amiodarone-mediated toxicity and autophagy induction in primary human hepatocytes (PHHs) from different donors (*n* = 8). In line with our findings in HepG2 cells, we observed a significant (*p* < 0.01) increase of caspase-3/-7 activity in PHHs after 2 h of treatment with amiodarone (200 µM) compared to untreated controls (1549.3 ± 304.1 RLU vs. 315.6 ± 120.8 RLU). Thereafter, caspase activity again declined (Fig. 5a). Western blot analysis confirmed a significant increase of active caspase-3 in PHHs at 2 h of amiodarone treatment followed by a decline at 4–8 h (Fig. 5b). In addition, increased phosphorylation of ER-stress regulators, i.e., IRE1α and eIF2α, could be observed at 2 h of PHH treatment with amiodarone, followed by a decline at 4–8 h (Fig. 5c). Decreased ER stress was associated with an increase of autophagy at 4 h of amiodarone-treated PHHs indicated by a decline of p62/SQSTM1 (Fig. 5d).

## Discussion

Amiodarone is a frequently used antiarrhythmic drug known to induce acute and chronic liver injury (Grimaldi-Bensouda et al. 2018; Björnsson 2015). Due to its long half-life and lipophilic structure, it persists in the liver for longer periods. Liver toxicity may therefore occur even after discontinuation of the therapy (Brien et al. 1987). The mechanisms leading to amiodarone-mediated liver injury have not yet been fully elucidated.

In the current study, we observed significantly higher serum levels of caspase-cleaved K18 in patients with chronic heart failure treated with amiodarone compared to those without amiodarone treatment, suggesting that amiodarone induces apoptotic liver injury. In contrast, aminotransferase levels were within the normal range in both groups. The discrepancy between aminotransferases and this apoptosis marker might be explained by a presumably higher sensitivity of the M30 assay and stability of the K18 fragments (Bantel et al. 2004; Feldstein et al. 2009; Olofsson et al. 2007; Cummings et al. 2006). Serological detection of caspase-cleaved K18 therefore allows the early detection of apoptotic liver injury despite normal aminotransferases, as shown in previous studies (Bantel et al. 2004; Kronenberger et al. 2005). Moreover, amiodarone-treated patients revealed mean serum levels of caspase-cleaved K18 > 200 U/L, a cut-off value which was closely related to the presence of non-alcoholic steatohepatitis (NASH) and fibrosis in NAFLD studies (Liebig et al. 2019; Feldstein et al. 2009). In contrast, patients without amiodarone treatment showed mean serum levels of caspase-cleaved K18 < 200 U/L, comparable to those of healthy individuals. Our in vitro analyses demonstrate that amiodarone indeed induced caspase-3/-7 activation in hepatocytes. Hepatocellular caspase activation was associated with TUNEL reactivity, indicating that cell death is mediated by apoptosis.

Since amiodarone is known to induce liver steatosis which sensitizes hepatocytes for apoptosis (Rabinowich and Shibolet 2015; Malhi et al. 2007; Vitins et al. 2014), we asked whether amiodarone-induced apoptosis is associated with lipid accumulation. We observed that amiodarone-mediated hepatocellular steatosis occurred earlier than caspase-3/-7 activation, suggesting that amiodarone-mediated lipid accumulation might trigger apoptosis. Amiodarone was shown to accumulate in hepatic mitochondria and to inhibit the electron transport chain (Fromenty et al. 1990a) which can result in ROS formation and apoptotic cell death. Amiodarone-mediated mitochondrial dysfunction also causes inhibition of β-oxidation of fatty acids which leads to triglyceride accumulation and subsequent liver steatosis (Fromenty et al. 1990b). Furthermore, lipid accumulation might be caused by amiodarone-induced expression of genes involved in lipogenesis (Anthérieu et al. 2011; Vitins et al. 2014). Indeed, we observed an increased mRNA expression of *SREBP1c*, a major transcription factor of de novo lipogenesis, as well as of DGAT1, an enzyme involved in triglyceride formation, in amiodarone-treated hepatocytes. Interestingly, transcripts for both regulators of lipogenesis were up-regulated by amiodarone in a similar time-dependent manner as observed for caspase activation.

Accumulated fatty acids trigger ER stress which leads to UPR activation (Cao et al. 2012). ER stress is not only a consequence, but can also cause lipid accumulation (Henkel and Green 2013). In this context, it could be demonstrated that *CHOP*-deficient mice revealed reduced amiodarone-mediated hepatic lipid accumulation and apoptosis (Erez et al. 2017). However, although pharmacologic inducers of ER stress such as tunicamycin cause liver steatosis in mice, they can suppress hepatic lipid synthesis suggesting that ER-stress-induced hepatic steatosis does not result from de novo lipogenesis (Erez et al. 2017; Rutkowski et al. 2008). Instead, ER stress has been shown to inhibit VLDL secretion, thereby contributing to lipid accumulation (Ota et al. 2008). In our study we could demonstrate that amiodarone triggers lipogenesis, indicated by up-regulation of *SREBP1c* and *DGAT1*, which was closely associated with lipid accumulation and activation of UPR branches (IRE1α, PERK/eIF2α) and consecutive *CHOP* induction in hepatocytes. CHOP can trigger apoptosis by up-regulation of pro-apoptotic molecules including TRAIL-R2, PUMA and BIM (Cazanave et al. 2011; Puthalakath et al. 2007; Jung et al. 2015). Vice versa, *CHOP*-deficient hepatocytes are protected from fatty acid-induced apoptosis (Cazanave et al. 2011). Furthermore, IRE1α-mediated activation of c-Jun-terminal kinase (JNK) leads to down-regulation of anti-apoptotic molecules and induction of BIM/BAX-mediated apoptosis (Lei and Davis 2003; Yamamoto et al. 1999; Rodriguez et al. 2012). JNK can also up-regulate TRAIL-R2 expression, thereby sensitizing hepatocytes for TRAIL-induced apoptosis (Malhi et al. 2007). ER stress associated apoptosis can therefore be mediated by the death receptor as well as by the mitochondrial death pathway.

In our study, we observed a decline of ER stress in the course of amiodarone treatment, as shown by decreased eIF2α phosphorylation and CHOP expression in hepatocytes. In accordance with declining ER stress, we found decreased caspase-3/-7 activation in amiodarone-treated hepatocytes. The reason for the time-dependent decline in amiodarone-mediated toxicity remains unclear. Recently, enhanced ER stress and liver injury have been associated with impaired autophagy in murine and human NAFLD which, conversely, could be reduced by autophagy induction (González-Rodríguez et al. 2014; Tanaka et al. 2016; Lin et al. 2013). Accordingly, mice with hepatocellular autophagy (*ATG7*)-deficiency revealed increased oxidative stress-induced activation of JNK and caspases as well as enhanced hepatocyte apoptosis (Wang et al. 2010; Amir et al. 2013; Römermann et al. 2020). Vice versa, treatment of patients with fibrinogen storage disease, which is characterized by the accumulation of fibrinogen aggregates and enhanced ER stress in the liver, with the autophagy inducer carbamazepine resulted in increased autophagy in the liver and decreased serum levels of caspase-cleaved K18 (Puls et al. 2013). Developing of strategies to minimize the risk of DILI is of great importance (Walker et al. 2020).

We therefore asked whether autophagy induction in the course of amiodarone treatment might counter-regulate its liver toxicity. Indeed, autophagy was induced in HepG2 cells and most strongly pronounced at 8 h of amiodarone treatment, paralleled by decreased ER-stress and caspase-3 activation. Vice versa, inhibition of autophagy by chloroquine resulted in enhanced caspase-3 activation in amiodarone-treated hepatocytes. The protective role of autophagy was further underlined by our observation that amiodarone-mediated hepatocellular toxicity was significantly increased in HepG2 cells deficient for the autophagy genes *ATG5* or *ATG7*. Similarly, it was demonstrated that autophagy inhibition increased amiodarone-mediated apoptosis in lung epithelial cells (Lee et al. 2013). Our data therefore suggest that amiodarone-mediated toxicity can be counter-regulated by autophagy induction. Amiodarone-mediated autophagy induction has been associated with ROS-induced adenine monophosphate-activated protein kinase (AMPK) activation, which in turn leads to inhibition of mTOR, a negative regulator of autophagy (Zhang et al. 2009; Choi et al. 2001). In this context, it is interesting to note that autophagy induction by the mTOR inhibitor rapamycin protects against apoptosis and acetaminophen-induced liver toxicity (Ravikumar et al. 2006; Ni et al. 2012). Pharmacological promotion of autophagy might therefore represent a promising therapeutic strategy to alleviate drug-induced liver injury.

## Abbreviations

ALT: Alanine aminotransferase
AST: Aspartate aminotransferase
DILI: Drug-induced liver injury
K18: Keratin-18
NAFLD: Non-alcoholic fatty liver disease
NASH: Non-alcoholic steatohepatitis
PHH: Primary human hepatocyte
RLU: Relative light units
ROS: Reactive oxygen species
UPR: Unfolded protein response
